# Health promotion of long-haul truck drivers and their families

**DOI:** 10.1590/0034-7167-2023-0511

**Published:** 2024-09-30

**Authors:** Fernanda Lise, Eda Schwartz

**Affiliations:** I Oregon State University. Corvallis, Oregon, United States of America; II Universidade Federal de Pelotas. Pelotas, Rio Grande do Sul, Brazil.; III Fundação Universidade Rio Grande. Rio Grande, Rio Grande do Sul, Brazil.

**Keywords:** Work-Life Balance, Family Health, Noncommunicable Diseases, Sustainable Development, Information Technology, Equilibrio entre Vida Personal y Laboral, Salud de la Familia, Enfermedades no Transmisibles, Desarrollo Sostenible, Tecnología de la Información

## Abstract

**Objectives:**

to describe the development of a Health Information and Communication Technology for the health care of long-haul truck drivers and their families.

**Methods:**

this is a description of the development of an Information and Communication Technology, developed from March to September 2023, following the systematization of the experience in five steps: 1) starting point, 2) initial questions, 3) recovery of the lived process, 4) background reflection, and 5) arrival points.

**Results:**

the technology called “Work-Family Balance,” electronically available, presents resources for the health care of long-haul truck drivers. It is anchored in studies on the health of long-haul truck drivers, notes from the International Labor Organization, the Strategic Action Plan for Confronting Chronic Diseases and Non-Communicable Diseases in Brazil, 2021-2030, and the Declaration by the International Association of Family Nursing.

**Final Considerations:**

the theoretical improvement of nursing can potentially improve the health care of long-haul truck drivers, prevent Chronic Non-Communicable Diseases, and promote work-life balance to achieve the goals of Agenda 2030.

## RESUMEN

**Objetivos:**

describir el desarrollo de una Tecnología de Información y Comunicación en Salud para el cuidado de la salud de los conductores de camiones de larga distancia y de sus famílias.

**Métodos:**

se trata de una descripción del desarrollo de una Tecnología de Información y Comunicación, realizada en el período de mayo a septiembre de 2023, siguiendo la Sistematización de la Experiencia en cinco etapas: 1) punto de partida, 2) preguntas iniciales, 3) recuperación del proceso vivido, 4) reflexión de fondo y 5) puntos de llegada.

**Resultados:**

la tecnología denominada “Equilibrio Trabajo-Familia,” disponible electrónicamente, presenta recursos para la atención a la salud de los conductores de camiones de larga distancia, anclada en estudios sobre la salud de estos conductores; Notas de la Organización Internacional del Trabajo; el Plan de Acciones Estratégicas para el Enfrentamiento de las Enfermedades Crónicas y Agravios No Transmisibles en Brasil, 2021-2030, y la declaración de la Asociación Internacional de Enfermería de Familias.

**Consideraciones Finales:**

el mejoramiento teórico de la enfermería tiene el potencial de mejorar la atención a la salud de los conductores de camiones de larga distancia, prevenir Enfermedades Crónicas No Transmisibles y promover el equilibrio trabajo-vida para alcanzar las metas de la Agenda 2030.

## INTRODUCTION

The Long-Haul Truck Driver’s (LHTD) work process involves several occupational stressors in the transportation industry, including long journeys, irregular working hours, and chronic stress^(1, 2, 3)^. These factors place LHTD driving among the most unhealthy and unsafe occupations, accentuated by the vulnerability to infection and transmission of the New Coronavirus. Given the syndemic caused by the SARS-CoV-2 pandemic, COVID-19 has both aggravated and been aggravated by other epidemics of Chronic Noncommunicable Diseases (NCDs) resulting from cardiovascular diseases such as hypertension, diabetes, obesity, smoking, and depression^(4)^.

Moreover, the lack of work-life balance increases conflicts between these two spheres and places work as an essential social determinant of health. In this sense, work-life balance refers to the time available for professional and personal activities involving other social roles, family activities, leisure activities, and other promoters of well-being and health, in addition to labor^(1)^.

Faced with this reality and considering that there is still a gap in the literature regarding nursing attention to the health of LHTD and their families, it is understood that equipping nursing with theoretical resources is a way to foster advances in health promotion. This aligns with the goals of the United Nations (UN) Agenda 2030^(5)^ for sustainable development, especially regarding objective number three, which focuses on the need to reduce mortality from NCDs, and objective number eight, which addresses the need to protect labor rights and promote healthy working environments.

From the above, it is essential to provide nursing with theoretical resources to improve the approach to the health of LHTD and their families, aiming at the prevention of chronic diseases and the promotion of work-life balance through the use of communication technologies in health. Internet sites are suitable spaces to conduct health education because of the availability of scientific evidence, documents, and recommendations endorsed by international organizations. These resources promote advances in nursing care technologies, which are crucial to achieving the 2030 Agenda targets for sustainable development^(5)^.

## OBJECTIVES

To describe the development of Information and Communication Technology for the health care of long-haul truck drivers and their families.

## METHODS

This is a description of the process of developing an Information and Communication Technology composed of studies on the health of long-haul truck drivers, available on the website https://cuidadoscomfamilias.com. To systematize the experience, conduct theoretical reflection, and promote change in practices, this report is supported by the process of systematization of experience by Oscar Jara Holliday^(6)^. Experience Systematization (ES) is composed of five steps: 1) the starting point; 2) the initial questions; 3) recovery of the lived process; 4) reflection in depth; 5) arrival points. These steps were adapted to the methodological study^(7)^. Phase 1 was an exploratory study involving the elaboration of a situational diagnosis and a review of evidence in the literature, and phase 2 involved the elaboration of ICT content. The study met the items of the Standards for Reporting Qualitative Research checklist: A Synthesis of Recommendations for qualitative studies^(8)^.

The process of development of Information and Communication Technology (ICT) was supported by literature review and other documents guiding nurses practice^(1, 2, 3, 5, 9, 10, 11)^ available on the https://cuidadoscomfamilias.com website. As for ethical aspects, this study reflects the opinions of the authors published in scientific studies. As it did not require any form of data collection, there was no need to submit to the research ethics committee for using public access information and for reviewing the literature, according to Resolution number 510 of the Brazilian National Health Council of 2016.

## RESULTS

### The First Step

This is the first step in the process of systematization of experience and involves participation in its development^(6)^. The Information and Communication Technology entitled “Work-Family Balance” had its conception influenced by the previous experiences of the authors in the research group NUCCRIN (Nucleus of Chronic Conditions and its Interfaces) and in research with patients on hemodialysis. These experiences provided evidence that several people had been long-haul truck drivers before hemodialysis treatment and that the main reasons for the development of chronic kidney disease (CKD) were Diabetes Mellitus (DM) and Systemic Arterial Hypertension (SAH) without follow-up and/or clinical control. This fact is corroborated by evidence from the literature, which also demonstrates the gap in the health care of LHTD and their families and the need to equip nurses to prevent and care for people with chronic diseases.

The content presented addresses the health of long-haul truck drivers and their families and is part of the post-doctoral study abroad (PDE) of the first author, developed at the School of Public Health of Oregon State University, supported by CNPq (National Council for Scientific and Technological Development), CNPq No. 26/2021, entitled “Evaluation of the Influence of Work-Life Balance on the Health of Long-Haul Truck Drivers in Times of Pandemic: Multicenter Study”.

### The Initial Questions

The second step resulted from questions that helped to delimit the object and the objective. According to Holliday, the initial questions involve questions such as: what do we want to systematize, what experiences do we want to systematize, and what central aspects of these experiences interest us to systematize^(6)^? This experience described meets a need identified in practice and literature. The development of the Information and Communication Technology entitled “Work-Family Balance” aims to improve the health care of long-haul truck drivers and the prevention of NCD. Its presentation collaborates in translating knowledge by promoting the transfer of knowledge primarily available in English, from the dissemination of synthesized information to nurses in Portuguese, contributing to the implementation of evidence in practice.

In addition, it meets an essential need for agile and viable communication tools that can be accessed by nurses and drivers from anywhere with internet access. It is an answer to the need for information for the assessment and health intervention of long-haul truck drivers and their families and the global challenges of the UN Agenda 2030 for sustainable development, in Objectives 3 [To reduce premature mortality by NCD by 1/3, via prevention and treatment, and promote mental health and well-being]; and 8 [To protect labor rights and promote safe and protected work environments for all workers in precarious jobs]^(5)^.

### The Recovery of the Lived Process

The third step involves rebuilding the process^(6)^. Initially, the problem related to the illness of drivers by NCD and the difficulties of access to health services were identified from data collection for research with Brazilian and foreign LHTDs, which started in March 2023 in southern Brazil. The development took place through the adaptation of the steps of the methodological study in two stages^(7)^: phase 1, an exploratory research phase involving the elaboration of a situational diagnosis and evidence review in the literature, and phase 2, the process of elaborating the content of the ICT.

In phase 1, a literature review was carried out in the databases PubMed/MEDLINE, LILACS, BIREME, and in free searches, aiming to answer the question: “What are the influences of professional life on the health of LHTD and the relationship with their family in times of pandemic?”. The objective was to understand the scientific production to support the practice of nurses in addressing the health of LHTD. The results were then categorized, evaluated, discussed, and interpreted. Phase 2 involved defining that information would be made available in thematic categories, with the possibility of comments and the incorporation of media such as images, photos, and links to other web pages or blogs to reference the contents. International studies and documents guiding practice were presented in electronic format, with the possibility of interaction between users who access the webpage and the https://cuidadoscomfamilias.com team.

To make the information available to nurses, we opted for the website Family Care, created in 2017 to offer students and nursing professionals classical literature and current scientific evidence essential for the practices of nurses, educators, and researchers, as well as a more appropriate approach to family health. The Family Care site was recognized by the Federal Nursing Council and the PAHO/WHO Collaborating Center of USP as a Nursing Now Brazil initiative (2021) (https://cuidadoscomfamilias.com/). This experience has contributed to disseminating advances in studies so that knowledge reaches the community, not only in Brazil, since this site is globally accessible. The number of visitors from abroad has increased over the years, reaching more than 2,500 accesses from the United States, Portugal, China, Uruguay, and Canada, among others.

**Figure 1:**
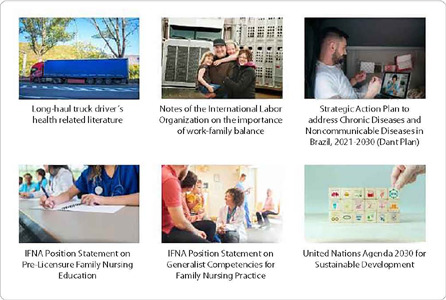
Presentation of the content available on the https://cuidadoscom-familias.com website

This technology is supported by current health-related LHTD literature,1-3 Agenda 2030 targets for sustainable development^(5)^, International Labour Organization (ILO) notes on the importance of work-family balance^(9)^, the Strategic Action Plan to Combat Chronic Diseases and Non-Communicable Diseases in Brazil, 2021-2030 (Dant Plan)^(10)^, and the position statements of the International Association of Family Nurses (IFNA) on family nursing education in the Bachelor of Nursing and on the competencies of the generalist nurse in the practice of family care^(11)^, with the possibility of interaction between users and staff, available at the electronic address (https://cuidadoscomfamilias.com) (Figure 1).

### Reflection

The fourth step involves the analysis, synthesis, and critical interpretation of the experience^(6)^. The development of the ICT entitled “Work-Family Balance” allowed us to understand the numerous challenges associated with the work process and life of LHTD. These challenges include difficulties in accessing health services, poor diet, lack of opportunities for physical activity, and the main factors associated with the exponential increase in the risk of cardiometabolic disease, cardiovascular disease, and chronic kidney disease, all of which result from the lack of control of Diabetes Mellitus (DM), systemic arterial hypertension (SAH), cholesterol, and obesity. As a synthesis, we highlight the lack of studies in the Portuguese language, which can be a barrier, considering that most scientific articles are in English. Thus, providing resources and interaction spaces for nurses and drivers is understood to be a way to contribute to promoting health care for LHTD and their families.

### The Arrival Points

The fifth step involves drawing conclusions and communicating learning^(6)^. It is concluded that the Information and Communication Technology: Work-Family Balance is an essential tool because it aims to equip nurses with scientific evidence to improve the approach to the health of LHTD and their families, and to reduce the risk factors of NCDs related to the work process, demographic, epidemiological, and nutritional transitions. It aims to achieve the goals of the UN Agenda 2030 for sustainable development, ensuring access to quality health and promoting well-being for all^(5)^.

Thus, the information and space made available through ICT can function as an e-Health Model to improve the monitoring, management, and quality of health care for individuals and their families. In addition, it can contribute to the reflection on the need for transformation in culture and the work process that can provide opportunities for behavioral changes, healthy habits, reduction of physiological and psychological stressors, and the achievement of work-life balance. This is an innovative, sustainable initiative concerned with human actions toward the environment and the well-being of people.

As a point of arrival, it is important to point out that this is not the endpoint. With the technology in use and constant updating, future steps will involve validation by nurses, health services, institutions representing road transport workers, and LHTD and their families.

## DISCUSSION

The literature that composes the Information and Communication Technology entitled “Health Care of Long-Haul Truck Drivers: Work-Family Balance and NCD Prevention” brings together international studies addressing issues related to access to health services and risk factors for NCDs such as Diabetes Mellitus^(2)^, Systemic Arterial Hypertension, stress at work, unhealthy eating, smoking, physical inactivity, overweight/obesity, obstructive sleep apnea^(3)^, prevention of sexually transmitted infections, mental health, and stress management^(1, 3)^. It also covers the relationship between stress, fatigue, and accidents^(3)^.

The high rate of NCDs contributes to the loss of years due to disability, according to Disability Adjusted Life Years (DALY), and results in significant damage to the health, social security, and economic systems of countries. World Bank analysis estimates that countries such as Brazil, China, India, and Russia lose 20 million productive years of life per year due to NCDs. The strategies for health promotion, comprehensive health care, health surveillance, and prevention of diseases and health problems are described in the Strategic Action Plan to Combat Chronic Diseases and Non-Communicable Diseases in Brazil, 2021-2030 (Dant Plan), emphasizing the need to qualify health professionals to disseminate good practices for the prevention of NCDs^(10)^. It also highlights the importance of changes in productive processes, management and organization of work, and work environments as strategies to promote worker health and safety at work.

The International Labour Organization (ILO) notes on work and family are based on the program’s Work and Family Information Sheets series about work and employment^(9)^. According to the ILO, work-life balance can suffer due to the need for time dedication, a scarce asset, to work-related commitments, which is essential for the survival of societies. The importance of discussion and reflection is highlighted so that governments take measures to change the model to reconcile work tasks and family responsibilities, which are invisible to the statistics of countries’ economies because it is an unpaid activity, despite its generated value^(9)^.

As a result of long working hours away from home, LHTDs may suffer from the lack of family presence, losing opportunities for more effective participation in the education of their children and in other private life activities and responsibilities, typical of workers who devote many hours to work. Work-family reconciliation measures involve organizing work processes that facilitate workers’ daily lives, such as organizing working time with flexible hours, providing information and training on company policies for family responsibilities, and additional leave as provided for in the constitution^(9)^.

Given this, governments have a fundamental role in the development of public policies that establish a legal framework and promote the reconciliation between work and family, allowing the abandonment of the culture of long working hours. The reduction of extended work hours increases productivity and reduces tiredness and accidents^(9)^, which can be very favorable to truck drivers, whose dedication to work often exceeds 10 hours a day, and especially to workers whose routes do not allow them to return home for weeks. Additionally, the ILO recommends that workers have their rights guaranteed for annual leave, emergency leave, parental leave, and maternity leave. It also ensures that the international labor standards laid down by ILO Convention number 189, which advocate for decent, productive work exercised in conditions of freedom, equality, security, and dignity, are met. Therefore, unions play a fundamental role in ensuring the implementation of legislation and advocating for improvements in public policies that promote work-family balance^(9)^.

The family is a fundamental aspect of any person’s life, and to promote families’ health, it is necessary to understand education and health practices. Therefore, it is considered essential that nurses know and support their practices in accordance with the International Association of Family Nurses (IFNA) statements on family nursing education and the competencies of the generalist nurse in the practice of family care^(11)^. Considering the reciprocal relationship between family and disease, both influence each other, and there is a need to understand family-centered theories involving the reciprocal relationship between individuals, family, community, health, and disease throughout evidence-based training.

The IFNA’s mission is to transform family health by serving as a unifying force and voice for family nursing globally, sharing knowledge, practices, and skills to enhance and nurture family nursing practice, and providing family nursing leadership through education, research, socialization, and collegial exchange in all aspects of family nursing^(11)^.

The ICT is an essential tool to improve the nursing approach to the health of LHTD and their families, reducing the risk factors of NCDs related to the work process and population aging, aiming to achieve the goals of the UN Agenda 2030 for sustainable development, which seeks to ensure access to quality health and promote well-being for all. As it is an innovative, sustainable initiative concerned with human actions towards the environment and the well-being of people, also can contribute to the reflection on the need for transformation in culture and the work process that can lead to behavioral changes, healthy habits, reduction of physiological and psychological stressors, and the achievement of work-life balance.

## FINAL CONSIDERATIONS

The development of Information and Communication Technology - Work-Family Balance, provided on the website https://cuidadoscomfamilias.com, aims to give visibility and improve the approach of nursing professionals to the health of truck drivers and their families. It also aims to promote advances in access to health services for this population, the prevention of NCDs, and reflection on the need for changes in the model of work organization and work-life balance.

The limitations of this study may be related to the sample of the studies used in the review, but this does not diminish the quality of the study. It should be noted that the studies available on the website https://cuidadoscomfamilias.com present evidence with the potential to support the practices of nurses in different contexts of action with LHTD and their families. Additionally, the recommendations of national and international organizations are considered generic and relevant to be used in different scenarios and cultures. They may support the development of other initiatives aimed at this population, serve as innovative resources for the prevention of NCDs, and contribute to achieving the objectives of Agenda 2030.
