# Supplementary material for: Health promotion of long-haul truck drivers and their families
Source: Rev Bras Enferm. 2024 Sep 30;77(Suppl 2):e20230511. doi: 10.1590/0034-7167-2023-0511 (PMC11441754; doi:10.1590/0034-7167-2023-0511)
Supplement: Translation

# Translation: Promoção da saúde de motoristas de caminhão de longa distância e suas famílias

**DOI:** 10.1590/0034-7167-2023-0511pt

**Authors:** Fernanda Lise, Eda Schwartz

**Affiliations:** I Oregon State University. Corvallis, Oregon, Estados Unidos da América.; II Universidade Federal de Pelotas. Pelotas, Rio Grande do Sul, Brasil.; III Fundação Universidade Rio Grande. Rio Grande, Rio Grande do Sul, Brasil.

**Keywords:** Equilíbrio Trabalho-Vida, Saúde da Família, Doenças Crônicas Não Transmissíveis, Objetivos do Desenvolvimento Sustentável, Tecnologia da Informação

## Abstract

**Objetivos:**

descrever o desenvolvimento de uma Tecnologia da Informação e Comunicação em Saúde para o cuidado da saúde dos motoristas de caminhão de longa distância e de suas famílias.

**Métodos:**

trata-se de uma descrição do desenvolvimento de uma Tecnologia da Informação e Comunicação, realizada no período de maio a setembro de 2023, seguindo a Sistematização da Experiência, em cinco etapas 1) ponto de partida, 2) perguntas iniciais, 3) recuperação do processo vivido, 4) reflexão de fundo e 5) pontos de chegada

**Resultados:**

a Tecnologia denominada Equilíbrio trabalho-família, disponibilizada eletronicamente, apresenta recursos para o a atenção à saúde de motoristas de caminhão de longa distância, ancorada em estudos sobre a saúde dos motoristas de caminhão de longa distância; Notas Organização Internacional do Trabalho; Plano de Ações Estratégicas para o Enfrentamento das Doenças Crônicas e Agravos Não Transmissíveis no Brasil, 2021-2030, e declaração da Associação Internacional de Enfermagem de famílias.

**Considerações Finais:**

o aprimoramento teórico da enfermagem tem potencial para melhorar a atenção à saúde dos motoristas de caminhão de longa distância, prevenir Doenças Crônicas Não Transmissíveis e promover o equilíbrio trabalho-vida para o alcance das metas da Agenda 2030.

## INTRODUÇÃO

O processo de trabalho do motorista de caminhão de longa distância envolve uma série de estressores ocupacionais que fazem parte da indústria de transporte e incluem, além de longas jornadas, horários de trabalho irregulares e estresse crônico^(1, 2, 3)^. Esses fatores colocam a condução de caminhões de longa distância entre as ocupações mais insalubres e inseguras, o que foi acentuado pela vulnerabilidade à infecção e transmissão do Novo Coronavírus. Diante da sindemia causada pela pandemia de SARS-CoV-2, a COVID-19 agravou e foi agravada por outras epidemias de Doenças Crônicas Não Transmissíveis (DCNTs) decorrentes de doenças cardiovasculares como hipertensão, diabetes, obesidade, tabagismo e depressão^(4)^.

Além disso, a falta de equilíbrio entre trabalho e vida aumenta os conflitos entre essas duas esferas e coloca o trabalho como um importante determinante social da saúde. Nesse sentido, o equilíbrio trabalho-vida diz respeito ao tempo disponível para as atividades profissionais e pessoais, envolvendo outros papéis sociais, atividades familiares, de lazer e outras promotoras de bem-estar e saúde, além das laborais^(1)^.

Frente a essa realidade e considerando que ainda existe uma lacuna na literatura em relação à atenção da Enfermagem à saúde dos motoristas de caminhão de longa distância e sua família, entende-se que instrumentalizar a enfermagem com recursos teóricos é uma forma de fomentar avanços na promoção da saúde e vai ao encontro das metas da Agenda 2030 da Organização das Nações Unidas (ONU)^(5)^ para o desenvolvimento sustentável, especialmente com relação aos seus objetivos número três, referente à necessidade de redução da mortalidade por doenças crônicas não transmissíveis, e ao número oito, relacionado à necessidade de proteção dos direitos trabalhistas e à promoção de ambientes de trabalho saudáveis.

A partir do exposto, denota-se a importância de disponibilizar à enfermagem recursos teóricos para melhorar a abordagem à saúde dos motoristas de caminhão de longa distância e sua família, visando a prevenção de doenças crônicas e a promoção do equilíbrio trabalho-vida, a partir do uso de tecnologias de comunicação em saúde. Tendo em vista que os sites da internet são espaços oportunos para realizar educação em saúde a partir da disponibilização de evidências científicas, documentos e recomendações chanceladas por organizações internacionais, é fundamental promover avanços nas tecnologias de cuidado da enfermagem, as quais são essenciais para o alcance das metas da Agenda 2030 para o desenvolvimento sustentável^(5)^.

## OBJETIVOS

Descrever o desenvolvimento de uma Tecnologia da Informação e Comunicação para o cuidado da saúde dos motoristas de caminhão de longa distância e de suas famílias.

## MÉTODOS

Trata-se de uma descrição do processo de desenvolvimento de uma Tecnologia da Informação e Comunicação composta por estudos sobre a saúde dos motoristas de caminhão de longa distância, disponibilizada no site Cuidados com Famílias. Para sistematizar a experiência, conduzir a reflexão teórica e promover a mudança nas práticas, esse relato está apoiado no processo de Sistematização da Experiência (SE) de Oscar Jara Holliday^(6)^, composto por cinco etapas: O ponto de partida; As perguntas iniciais; Recuperação do processo vivido; A reflexão a fundo e Os pontos de chegada.

Para sua construção, foram adaptados os passos do estudo metodológico^(7)^. A fase 1 consistiu em um estudo exploratório com a elaboração de um diagnóstico situacional e revisão de evidências na literatura, e na fase 2 ocorreu o processo de elaboração do conteúdo da TIC. O estudo atendeu aos itens do checklist *Standards for Reporting Qualitative Research: A Synthesis of Recommendations* para estudos qualitativos^(8)^.

O processo de desenvolvimento da Tecnologia da Informação e Comunicação (TIC) foi apoiado em revisão de literatura e outros documentos norteadores da prática de enfermeiros(as)^(1, 2, 3, 5, 9, 10, 11)^, disponibilizados no site cuidadoscomfamilias.com. Quanto aos aspectos éticos, este estudo reflete as opiniões dos autores, publicadas em estudos científicos. Por não necessitar de nenhuma forma de coleta de dados, não houve necessidade de submissão ao comitê de ética em pesquisa, por utilizar informações de acesso público e de revisão de literatura, conforme a Resolução número 510 do Conselho Nacional de Saúde de 2016.

## RESULTADOS

### O ponto de partida

Esse é o primeiro passo do processo de sistematização da experiência e envolve a participação no seu desenvolvimento^(6)^. A Tecnologia da Informação e Comunicação intitulada “Equilíbrio Trabalho-Família” teve sua concepção influenciada pelas experiências anteriores das autoras no grupo de pesquisa NUCCRIN (Núcleo de Condições Crônicas e suas Interfaces) e em pesquisa com pacientes em hemodiálise. Essas experiências permitiram evidenciar que diversas pessoas haviam sido motoristas de caminhão de longa distância antes do tratamento de hemodiálise e que os principais motivos do desenvolvimento da Doença Renal Crônica (DRC) foram Diabetes Mellitus (DM) e Hipertensão Arterial Sistêmica (HAS) sem acompanhamento ou controle clínico. Fato corroborado por evidências da literatura, as quais demonstram ainda a lacuna na atenção à saúde dos motoristas de caminhão de longa distância e de suas famílias e a necessidade de instrumentalizar o enfermeiro para a prevenção e cuidado das pessoas com doenças crônicas.

O conteúdo apresentado aborda a saúde dos motoristas de caminhão de longa distância e de suas famílias e faz parte do estudo de pós-doutorado no exterior (PDE) da primeira autora, desenvolvido na Faculdade de Saúde Pública da *Oregon State University,* apoiado pelo CNPq (Conselho Nacional de Desenvolvimento Científico e Tecnológico), Chamada CNPq N.º 26/2021, intitulado “Avaliação da Influência do Equilíbrio Trabalho-Vida na Saúde de Motoristas de Caminhão de Longa Distância em Tempos de Pandemia: Estudo Multicêntrico”.

### As perguntas iniciais

O segundo passo resultou de questionamentos que ajudaram a delimitar o objeto e o objetivo. Segundo Holliday, as perguntas iniciais envolvem indagações como: para que queremos sistematizar, que experiências queremos sistematizar e quais aspectos centrais dessas experiências nos interessam sistematizar^(6)^? Essa experiência descrita atende a uma necessidade identificada na prática e na literatura. O desenvolvimento da Tecnologia da Informação e Comunicação intitulada “Equilíbrio Trabalho-Família” tem como objetivo melhorar a atenção à saúde de motoristas de caminhão de longa distância e a prevenção de DCNT. Sua apresentação colabora na tradução do conhecimento por promover a transferência do conhecimento disponível, em sua maioria, no idioma inglês, a partir da difusão e disseminação de informações sintetizadas aos enfermeiros, em português, contribuindo para a implementação das evidências na prática.

Além disso, atende a uma importante necessidade de ferramentas de comunicação ágeis e viáveis, possíveis de serem acessadas por enfermeiros(as) e motoristas de qualquer lugar com acesso à internet. Trata-se de uma resposta para atender a necessidade de informações para a avaliação e intervenção em saúde dos motoristas de caminhão de longa distância e suas famílias e os desafios globais da Agenda 2030 da ONU para o desenvolvimento sustentável, nos objetivos 3 [reduzir em 1/3 a mortalidade prematura por DCNT, via prevenção e tratamento, e promover a saúde mental e o bem-estar] e 8 [proteger os direitos trabalhistas e promover ambientes de trabalho seguros e protegidos para todos os trabalhadores em empregos precários]^(5)^.

### A recuperação do Processo vivido

O terceiro passo envolve a reconstrução do processo^(6)^. Inicialmente, identificou-se na prática o problema relacionado ao adoecimento dos motoristas por DCNT, bem como as dificuldades enfrentadas por eles no acesso aos serviços de saúde. A partir da coleta de dados para a pesquisa com motoristas de caminhão de longa distância brasileiros e estrangeiros, iniciada em março de 2023, no Sul do Brasil, o desenvolvimento ocorreu entre maio e setembro de 2023, adaptando os passos do estudo metodológico em duas etapas^(7)^. A fase 1 consistiu em um estudo exploratório com a elaboração de um diagnóstico situacional e revisão de evidências na literatura, e na fase 2 ocorreu o processo de elaboração do conteúdo da TIC.

Na fase 1, realizou-se uma revisão de literatura nas bases de dados PubMed/MEDLINE, LILACS, BIREME, e em buscas livres, visando responder à questão: “Quais são as influências da vida profissional na saúde dos motoristas de caminhão de longa distância e na relação com a família, em tempos de pandemia?”. O objetivo era conhecer a produção científica para apoiar a prática de enfermeiros na abordagem à saúde desses motoristas. Procedeu-se à categorização, avaliação, discussão e interpretação dos resultados. A fase 2 envolveu as definições de que as informações seriam disponibilizadas em categorias temáticas, com possibilidade de comentários, e incorporação de mídias como imagens, fotos e links para outras páginas da web ou blogs para referenciar os conteúdos. Também se apresentou os estudos internacionais e documentos norteadores da prática em formato eletrônico, com a possibilidade de interação entre usuários que acessam a webpage e a equipe Cuidados com Famílias.

Para disponibilizar as informações aos enfermeiros, optou-se pelo site Cuidados com Famílias, criado em 2017 para oferecer aos estudantes e profissionais da Enfermagem a literatura clássica e evidências científicas atuais, essenciais para as práticas de enfermeiros(as), educadores e pesquisadores(as) na abordagem mais adequada à saúde das famílias. O site Cuidados com Famílias foi reconhecido pelo Conselho Federal de Enfermagem e pelo Centro Colaborador OPAS/OMS da USP como uma iniciativa *Nursing Now Brasil* (2021) (https://cuidadoscomfamilias.com/). Tal experiência tem contribuído para divulgar avanços dos estudos, de modo que o conhecimento chegue até a comunidade, não apenas no Brasil, tendo em vista que o referido site é de acesso global. O número de visitantes do exterior tem aumentado ao longo dos anos, alcançando mais de 2.500 acessos, oriundos dos Estados Unidos, Portugal, China, Uruguai e Canadá, entre outros.

Essa tecnologia está apoiada em literatura atual relacionada à saúde do motorista de caminhão de longa distância^(1, 2, 3)^; nas metas da Agenda 2030 para o desenvolvimento sustentável^(5)^; em notas da Organização Internacional do Trabalho (OIT) sobre a importância do equilíbrio entre trabalho-família^(9)^; no Plano de Ações Estratégicas para o Enfrentamento das Doenças Crônicas e Agravos Não Transmissíveis no Brasil, 2021-2030 (Plano de Dant)^(10)^; e nas declarações de posicionamento da Associação Internacional de Enfermeiros de Famílias (IFNA) sobre educação em enfermagem de família na Graduação em Enfermagem e sobre as competências do enfermeiro generalista na prática de cuidados à família^(11)^, com a possibilidade de interação entre usuários e equipe, disponibilizados no endereço eletrônico (https://cuidadoscomfamilias.com/) do site Cuidados com Famílias (Figura 1).

**Figura 1:** Apresentação do conteúdo disponibilizado no site https://cuidadoscomfamilias.com

### Reflexão

O quarto passo envolve a análise, síntese e interpretação crítica da experiência^(6)^. A experiência de desenvolvimento da Tecnologia da Informação e Comunicação intitulada “Equilíbrio Trabalho-Família” permitiu compreender os inúmeros desafios associados ao processo de trabalho e vida do motorista de caminhão de longa distância. Especialmente, as dificuldades de acesso aos serviços de saúde, má alimentação, falta de atividade física, e como os principais fatores associados ao aumento exponencial do risco de doença cardiometabólica, cardiovascular e Doença Renal Crônica, decorrentes do descontrole dos níveis de Diabetes

Mellitus (DM), Hipertensão Arterial Sistêmica (HAS), colesterol e obesidade. Como síntese, destacamos a carência de estudos no idioma português, o que pode ser uma barreira, considerando que a maioria dos artigos científicos estão no idioma inglês. Dessa forma, entende-se que disponibilizar recursos e espaço de interação aos enfermeiros(as) e motoristas é uma maneira de contribuir na promoção da atenção à saúde do motorista de caminhão de longa distância e de sua família.

### Os pontos de chegada

O quinto passo envolve a formulação de conclusões e a comunicação da aprendizagem^(6)^. Conclui-se que a Tecnologia da Informação e Comunicação “Equilíbrio Trabalho-Família” é uma ferramenta importante, pois visa instrumentalizar os enfermeiros(as) com evidências científicas para melhorar a abordagem à saúde dos motoristas de caminhão de longa distância e de suas famílias, e diminuir os fatores de risco de DCNTs relacionadas ao processo de trabalho, à transição demográfica, epidemiológica e nutricional, visando o alcance das metas da Agenda 2030 da ONU para o desenvolvimento sustentável de garantir o acesso à saúde de qualidade e promover o bem-estar para todos^(5)^.

Desse modo, as informações e o espaço disponibilizado a partir da TIC podem funcionar como um modelo de eSaúde para melhorar o acompanhamento, o gerenciamento e a qualidade da atenção à saúde do indivíduo e de sua família. Além disso, pode contribuir para a reflexão sobre a necessidade de transformação na cultura e no processo de trabalho que possam oportunizar mudanças comportamentais, hábitos saudáveis, redução dos estressores fisiológicos e psicológicos, e o alcance do equilíbrio entre trabalho e vida, por se tratar de uma iniciativa inovadora, sustentável, preocupada com as ações humanas ao meio ambiente e no bem-estar das pessoas.

Como ponto de chegada, é importante salientar que esse não é o ponto final. Estando a tecnologia em uso e constante atualização, os passos futuros apresentarão a validação por enfermeiros, serviços de saúde e de instituições representantes dos trabalhadores em transporte rodoviário, e pelos motoristas de caminhão de longa distância e suas famílias.

## DISCUSSÃO

A literatura que compõe a Tecnologia da Informação e Comunicação intitulada Cuidado à saúde de motoristas de caminhão de longa distância: Equilíbrio trabalho-família e prevenção de DCNT reuniu estudos internacionais que abordam temas relacionados ao acesso aos serviços de saúde, risco para doenças crônicas não transmissíveis como Diabetes Mellitus (DM)^(2)^, Hipertensão Arterial Sistêmica (HAS), estresse no trabalho, alimentação não saudável, tabagismo, inatividade física, sobrepeso/obesidade, apneia obstrutiva do sono^(3)^, Prevenção de 1ST (Infecção Sexualmente Transmissível), saúde mental, gerenciamento do estresse^(^1,3^)^, relação entre estresse, fadiga e acidentes^(3)^.

O alto índice de Doenças Crônicas Não Transmissíveis contribui para a perda de anos por incapacidade, conforme o DALY (*Disability Adjusted Life Years*) e prejuízos aos sistemas de saúde, de seguridade social e da economia dos países. Análise do Banco Mundial estima que países como o Brasil, China, Índia e Rússia perdem, anualmente, 20 milhões de anos produtivos de vida pelas DCNT. As estratégias para promoção da saúde, atenção integral à saúde, vigilância em saúde e prevenção a doenças e agravos à saúde estão descritas no Plano de Ações Estratégicas para o Enfrentamento das Doenças Crônicas e Agravos Não Transmissíveis no Brasil, 2021-2030 (Plano de Dant), ressaltando a necessidade de qualificação dos profissionais de saúde para disseminar boas práticas para prevenção das DCNT^(10)^. Assim como, a importância de mudanças nos processos produtivos, nas formas de gestão e organização do trabalho e nos ambientes de trabalho como estratégia da promoção de saúde do trabalhador e segurança no trabalho^(10)^.

As notas da Organização Internacional do Trabalho (OIT) sobre trabalho e família baseiam-se na série *Work and Family Information Sheets* do Programa sobre Condições de Trabalho e Emprego (TRAVAIL)^(9)^. De acordo com a OIT o equilíbrio trabalho-vida pode sofrer prejuízos diante das necessidades de dedicação de tempo, um bem escasso, aos compromissos relacionados ao trabalho, o qual é uma necessidade para a sobrevivência das sociedades. Posto isso, denota-se a importância da discussão e reflexão para que medidas sejam tomadas pelos governos para a mudança de modelo que vise a conciliação entre as tarefas do trabalho e as responsabilidades familiares, que são invisíveis às estatísticas da economia dos países por ser uma atividade não remunerada, apesar do valor gerado^(9)^.

Em consequência das longas jornadas de trabalho fora de casa, o motorista de caminhão de longa distância pode sofrer com a falta da família, ter perdas de oportunidade de participação mais efetiva na educação dos filhos e em outras atividades e ou responsabilidades da vida privada, características de trabalhadores que dedicam muitas horas ao trabalho. As medidas de conciliação entre trabalho e família envolvem organização de processos de trabalho que facilitem o cotidiano de trabalhadores e trabalhadoras, como a organização do tempo de trabalho com horário flexível, serviço de informação e formação sobre as políticas da empresa para responsabilidades familiares, licenças adicionais as previstas na constituição^(9)^.

À vista disso, os governos tem papel fundamental no desenvolvimento de políticas públicas que estabeleçam um marco legal e favoreçam a conciliação entre trabalho e família, permitindo abandonar a cultura de longas jornadas de trabalho, uma vez que a redução dos horários ampliados dedicados ao trabalho aumentam a produtividade, reduzem o cansaço e a ocorrência de acidentes^(9)^, o que pode ser muito favorável aos motoristas de caminhão, cuja dedicação ao trabalho ultrapassa 10 horas diárias e especialmente aos trabalhadores cuja rotas não permita retornar para casa durante semanas. Além disso, a OIT recomenda que os trabalhadores tenham seus direitos assegurados de férias anuais, licença por motivo de emergência, licença parental e a licença-maternidade. Assim como, garantia de que as normas internacionais do trabalho, estabelecidas pela Convenção no 189 da OIT, de trabalho decente, produtivo, exercido em condições de liberdade, igualdade, segurança e dignidade sejam cumpridas. Logo, os sindicatos têm papel fundamental na garantia a implementação da legislação e em advogar por melhorias nas políticas públicas que promovam equilíbrio entre trabalho e família^(9)^.

A família é um aspecto fundamental na vida de qualquer pessoa e para promover a saúde das famílias é necessário conhecer sobre educação e práticas em saúde da mesma. Por isso, considera-se primordial que os enfermeiros conheçam e apoiem suas práticas nas Declarações da Associação Internacional de Enfermeiros de Famílias (IFNA) sobre educação em enfermagem de família e sobre as Competências do enfermeiro generalista na prática de cuidados à família^(11)^. Considerando o relacionamento recíproco entre família e doença, posto que, ambos se influenciam e a necessidade de conhecer as teorias centradas na família envolvendo a relação recíproca entre indivíduos, família, comunidade, saúde e doença ao longo da formação baseada em evidência.

A IFNA tem a missão de transformar a saúde da família ao servir como uma força unificadora e voz para a enfermagem familiar globalmente, além de compartilhar conhecimentos, práticas e habilidades para aprimorar e nutrir a prática de enfermagem familiar; e fornecer liderança de enfermagem de família por meio de educação, pesquisa, socialização e intercâmbio colegial em todos os aspectos da enfermagem de família^(11)^.

A TIC é uma ferramenta essencial para melhorar a abordagem de enfermagem da saúde do LHTD e de suas famílias, reduzindo os fatores de risco das DNTs relacionadas ao processo de trabalho e ao envelhecimento da população, com o objetivo de atingir os objetivos da agenda da ONU 2030 para o desenvolvimento sustentável, que procura garantir o acesso a saúde de qualidade e promover o bem-estar para todos. Por ser uma iniciativa inovadora e sustentável preocupada com as ações humanas em relação ao meio ambiente e com o bem-estar das pessoas, também pode contribuir para a reflexão sobre a necessidade de transformação na cultura e no processo de trabalho que podem levar a mudanças comportamentais, hábitos saudáveis, redução de estressores fisiológicos e psicológicos e a conquista do equilíbrio entre vida profissional e pessoal.

## CONSIDERAÇÕES FINAIS

O desenvolvimento da Tecnologia da Informação e Comunicação - Equilíbrio trabalho-vida, disponibilizada no site Cuidados com Famílias visa contribuir para dar visibilidade e aprimorar a abordagem dos profissionais da enfermagem à saúde dos motoristas de caminhão e suas famílias e promover avanços no acesso dessa população aos serviços de saúde, a prevenção de doenças crônicas não transmissíveis, a reflexão sobre a necessidade de mudanças no modelo de organização de trabalho e o equilíbrio trabalho-vida.

As limitações deste estudo podem estar relacionadas a amostra dos estudos utilizados na revisão, o que não diminui a qualidade do estudo. Ressalta-se que os estudos disponibilizados no site Cuidados com Famílias apresentam evidencias com potencial de apoiar as práticas de enfermeiros (as) em diferentes contextos de atuação com motoristas de caminhão de longa distância e suas famílias. Além desses, as recomendações de organizações nacionais e internacionais são consideradas genéricas e pertinentes para serem utilizadas em diferentes cenários e ou culturas e poderão ser apoiar o desenvolvimento de outras inciativas destinadas a essa população e servir como recursos inovadores para a prevenção das DCNTs, assim como, contribuir para o alcance dos objetivos da Agenda 2030.
